# National Library and Museum—Dental

**Published:** 1896-05

**Authors:** 


### EDITORIAL.

### National Library and Museum.—Dental.

At the last annnal meeting of the American Dental Association a committee was appointed to take into consideration the matter of establishing a Dental Library and Museum in connection with the National Army Medical Museum and Library at Washington, D. C. This committee was asked to consider the subject in all its bearings, and were given power to act, so far, at least, as taking the initiative for carrying out the proposed object. This committee consists of Dr. Wm. Donnelly, of Washington, D. C.; Dr. J. Taft, of Cincinnati, O.; Dr. L. D. Shepherd, of Boston, Mass.; Dr. J. H. McKellops, of St. Louis, Mo. and Dr. Henry Morgan, of Nashville, Tenn. Some attention and thought has been bestowed upon the matter by the committee, and a circular to the profession has just been issued. This circular emphasizes the importance and value to the dental profession of such a national collection, which, when established, would serve for reference not only in regard to things present, but would especially illustrate the past history of the science and art of dentistry; and further than this, this department of the collection would, in common with others, have behind it the support of the Government, which will permanently maintain such a collection at the National Capital. At first this institution, namely, the Army Medical Museum and Library, was limited to military medical subjects, but later greatly broadened its scope until it is now practically a medical department of the Government, maintained by congressional appropriations, housed in a large building erected for the purpose, extended to cover the whole field of medicine and surgery, and open to the public the intellectual property of all classes and a nationalized collection for the medical profession. This vast museum is visited annually by over 50,000 persons and consulted by over 3,000 students. The museum contains about 35,000 specimens, of which over 12,000 are pathologic. The Army Medical Library is the largest and most complete of its kind in existence. It contains three-fourths of the medical literature of the world, and nine-tenths of that of the past ten years. It contains 120,000 bound volumes and 190,000 pamphlets. Its literature is greater in volume than the medical literature of either the library of the British Museum or the National Library of France. It covers a wide field, and embraces a better reference and working collection. It has an unequaled index catalogue of 18,000 pages. The dental section of this library contains a large and choice collection of the recent literature in English and other languages relative to dentistry, and our efforts will be to make this section complete as far as possible, especially embracing the rarer publications which disclose the conditions from which modern dentistry developed and revealed the history of the forces and factors operative in the evolution of the distinct profession.

While the library of national dental literature is being built up, the museum department should by no means be overlooked or neglected. Everything that can in any sense add to the interest of such a collection should ultimately be found in this department, such as instruments, models, drawings, machinery, also material of all sorts. The importance of prompt action in making such a collection of both books and specimens is especially emphasized, when it is remembered that as time goes on many things are continually passing out of reach. Early literature of the profession is becoming more and more rare every year, and is fast passing out of reach altogether. While it is true that some of the dental colleges of our country are becoming interested in making collections, in both of these directions, it is equally true that such collections, with however much of interest they may be gathered, can never take the place of, nor serve the purpose, of a national library and museum. So far as the schools are concerned their collections will be made with special reference to their use in their courses of instruction. That is about the only use a dental college could have in procuring and arranging a library. Those objects and books which could not be utilized in this way would necessarily be eliminated and most certainly lost. All specimens and books sent to the National Museum will be carefully arranged and labeled with the name of the contributor and so placed as to be easily accessible. Ample room is guaranteed for this collection. No one should hesitate to send anything fitted for such a collection because he might imagine there were duplicates already there. Indeed, we can conceive that one of the valuable points in such a collection would be the opportunity it would afford to dental colleges to make exchanges, or to procure things that could not elsewhere be found ; so do not hesitate to send because there might be multiplication of some objects. Another point to be borne in mind is that such a collection will be more permanent and enduring than any private collection could possibly be, and much more permanent thanit could be in a collection of a dental college.

"WHAT are you doing?" Dr. Holmes once asked a student in the dissecting-room. "Ligating arteries, sir." "Why not say tie?" rejoined the doctor. "I find that country practitioners ligate arteries and that surgeons tie them." The best of this anecdote is that the unappreciative student spread it as a joke against Dr. Holmes.—*Exchange.*

